# Supplementary figures and images for: A pooled‐sample draft genome assembly provides insights into host plant‐specific transcriptional responses of a Solanaceae‐specializing pest, Tupiocoris notatus (Hemiptera: Miridae)
Source: Ecol Evol. 2024 Mar 11;14(3):e10979. doi: 10.1002/ece3.10979 (PMC10928254; doi:10.1002/ece3.10979)

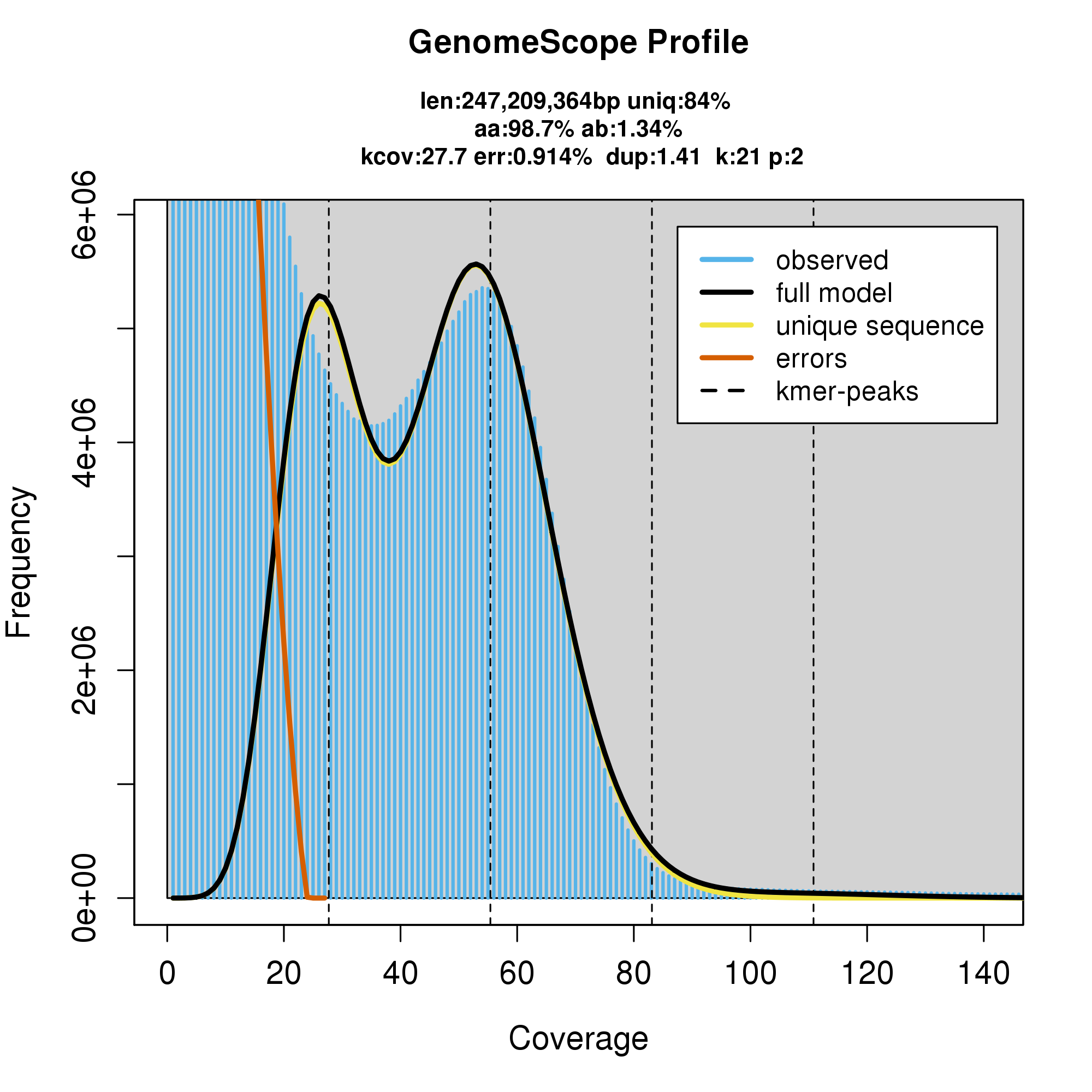

Supplement: Supplementary file 1 — Figure S1. [file ECE3-14-e10979-s004.png]

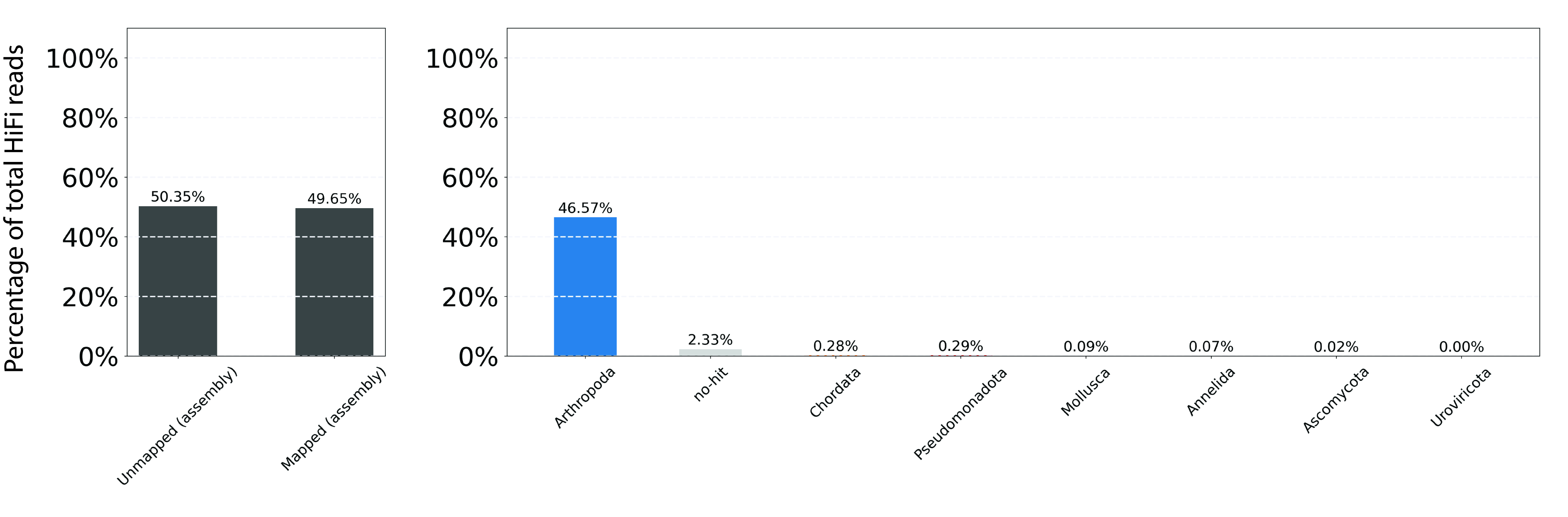

Supplement: Supplementary file 2 — Figure S2. [file ECE3-14-e10979-s009.tif]
